# Aphasic status epilepticus presenting with prominent psychiatric symptoms: A case report

**DOI:** 10.1002/pcn5.70440

**Published:** 2026-11-10

**Authors:** Yuto Yamada, Shigeru Takahashi, Masaki Fujiwara

**Affiliations:** ^1^ Department of Psychiatry Sekizen Hospital Tsuyama Japan; ^2^ Department of Neuropsychiatry Okayama University Graduate School of Medicine, Dentistry, and Pharmaceutical Sciences Okayama Japan; ^3^ Department of Neuropsychiatry, Medical Development Field Okayama University Okayama Japan

**Keywords:** agitation, aphasic status epilepticus, electroencephalogram, disease onset

## Abstract

**Background:**

Aphasic status epilepticus (ASE) is a subtype of nonconvulsive status epilepticus characterized by aphasia as its primary ictal manifestation. Although psychiatric symptoms are uncommon in ASE, they may obscure the diagnosis when present. We report an atypical case, presenting predominantly with agitation and irritability, which was rapidly diagnosed as ASE by electroencephalography (EEG) based on the abrupt symptom onset and neurological history.

**Case Presentation:**

A 49‐year‐old right‐handed woman with alcohol use disorder was referred to our psychiatric hospital because of agitation, irritability, and disorganized thinking. Approximately 2 months before presentation to our hospital, she developed a generalized tonic–clonic seizure 3 days after sustaining a head injury. A left posterior fossa subdural hematoma was diagnosed, and burr‐hole evacuation was performed at a general hospital. She continued rehabilitation; however, 6 days before admission to our hospital, she suddenly developed incoherent speech with agitation and irritability, prompting referral to our psychiatric hospital. Neurological examination revealed sensory aphasia. Given the history of brain injury and abrupt symptom onset, EEG was performed and demonstrated left‐dominant periodic lateralized epileptiform discharges, confirming ASE. Antiseizure treatment resolved the EEG abnormalities and aphasia; however, aphasia, agitation, and irritability recurred with periodic lateralized epileptiform discharges. The symptoms improved with intravenous diazepam, and no recurrence was observed for >2 years following optimization of antiseizure medication.

**Conclusion:**

ASE may present predominantly with psychiatric symptoms and be misdiagnosed as a primary psychiatric disorder. Recognizing symptom chronology and epileptogenic background is essential for prompting EEG evaluation and early diagnosis.

## BACKGROUND

Aphasia can occur as an ictal manifestation of epilepsy and, in rare cases, may be the predominant manifestation of status epilepticus.^1^, ^2^ Aphasic status epilepticus (ASE) is a subtype of nonconvulsive status epilepticus that is characterized by aphasia as the primary ictal manifestation; antiseizure treatment leads to resolution of the language disturbance.^1^ Because ASE may occur without prominent motor manifestations, electroencephalography (EEG) plays an important role in establishing the diagnosis and differentiating ASE from other causes of acute‐onset aphasia.^1^, ^3^ Although psychiatric symptoms are generally considered uncommon in patients with ASE.^2^ several cases presenting with prominent psychiatric manifestations have been reported,^4^, ^5^, ^6^ including a patient who was initially misdiagnosed with a primary psychiatric disorder.^7^ Here, we report an atypical case of ASE in which agitation and irritability were the predominant presenting symptoms, resulting in an initial failure to suspect an epileptic etiology. Prompt EEG, performed on the basis of the abrupt symptom onset and the patient's neurological history, led to the early diagnosis and successful treatment of ASE.

## CASE PRESENTATION

A 49‐year‐old right‐handed woman with a history of alcohol use disorder and alcoholic liver cirrhosis was referred to our psychiatric hospital because of agitation, irritability, and disorganized thinking. She had consumed alcohol daily for 12 years and subsequently developed alcoholic liver cirrhosis but had consistently refused psychiatric treatment. She had no history of other substance use disorders besides alcohol use disorder.

Approximately 2 months before presentation to our hospital, she fell at home while intoxicated and sustained a head injury. Three days later, she developed a generalized tonic–clonic seizure and was admitted to General Hospital A. Brain computed tomography revealed a left posterior fossa subdural hematoma and traumatic cerebral contusion, and burr‐hole evacuation of the hematoma was performed. She exhibited no psychiatric symptoms after surgery, and the seizure was considered an acute symptomatic seizure; antiseizure medication was therefore not initiated.

On Day 43 after the initial seizure, she was transferred to General Hospital B for rehabilitation. Her rehabilitation progressed uneventfully until Day 62, when she suddenly developed incoherent speech and was unable to name objects. At the same time, she became increasingly agitated and irritable, and wandered restlessly around the ward. Follow‐up head computed tomography revealed no new abnormalities, and EEG was not performed. Alcohol‐related psychiatric disorder or psychiatric sequelae of traumatic brain injury was suspected, and she was referred to our psychiatric hospital on Day 68 after the initial seizure.

At the initial psychiatric evaluation, her blood pressure was 128/74 mmHg, heart rate was 78 beats/min, respiratory rate was 16 breaths/min, body temperature was 37.6°C, and oxygen saturation was 98% on room air. No signs or symptoms of autonomic hyperactivity, such as nausea, tremor, sweating, or tachycardia, were observed. Neurological examination revealed no cranial nerve abnormalities including ophthalmoplegia, pyramidal signs, or focal motor deficits. However, the assessment of cerebellar function was difficult because of her behavioral symptoms. Accurate assessment of her level of consciousness was also difficult because of severe aphasia; however, she was awake with spontaneous eye opening and remained responsive to her surroundings and family members, which suggests that she was relatively alert. During the interview, she spoke continuously and maintained fluent speech; therefore, motor aphasia was not suspected. However, her utterances were fragmented and lacked meaningful content. She also demonstrated impaired repetition, suggesting sensory aphasia. Marked agitation and hyperactivity were observed; she was unable to remain seated, constantly looked around the room, and did not maintain attention during the interview.

Laboratory testing revealed no marked abnormalities except for a mildly elevated gamma‐glutamyl transferase level of 49 U/L (normal, 9–32 U/L). Head computed tomography demonstrated a low‐density lesion around the posterior horn of the lateral ventricle, which was considered a sequela of the traumatic cerebral contusion (Figure 1). No new intracranial lesions were observed. Given the history of traumatic brain injury and the abrupt symptom onset, an epileptic etiology was suspected, and EEG was performed. EEG demonstrated left‐dominant periodic lateralized epileptiform discharges (Figure 2). On the basis of these findings, ASE was suspected, and the patient was transferred to General Hospital A on the same day for further neurological management. Brain magnetic resonance imaging performed at General Hospital A revealed no additional abnormalities including hyperintense FLAIR/T2 signal in the thalami and periaqueductal gray matter (Figure 3). Because both the aphasia and psychiatric symptoms remained difficult to control, the patient was transferred to General Hospital C 6 days later, on Day 74 after the initial seizure. Perampanel (6 mg/day) was initiated, resulting in the disappearance of both the epileptiform discharges on EEG and the aphasic symptoms. However, treatment with risperidone (2 mg/day) for persistent agitation and irritability resulted in only limited improvement. On Day 128 after the initial seizure, she was readmitted to our hospital for the management of her residual psychiatric symptoms, rehabilitation for lower‐extremity weakness, and discharge planning.

**Figure 1:**
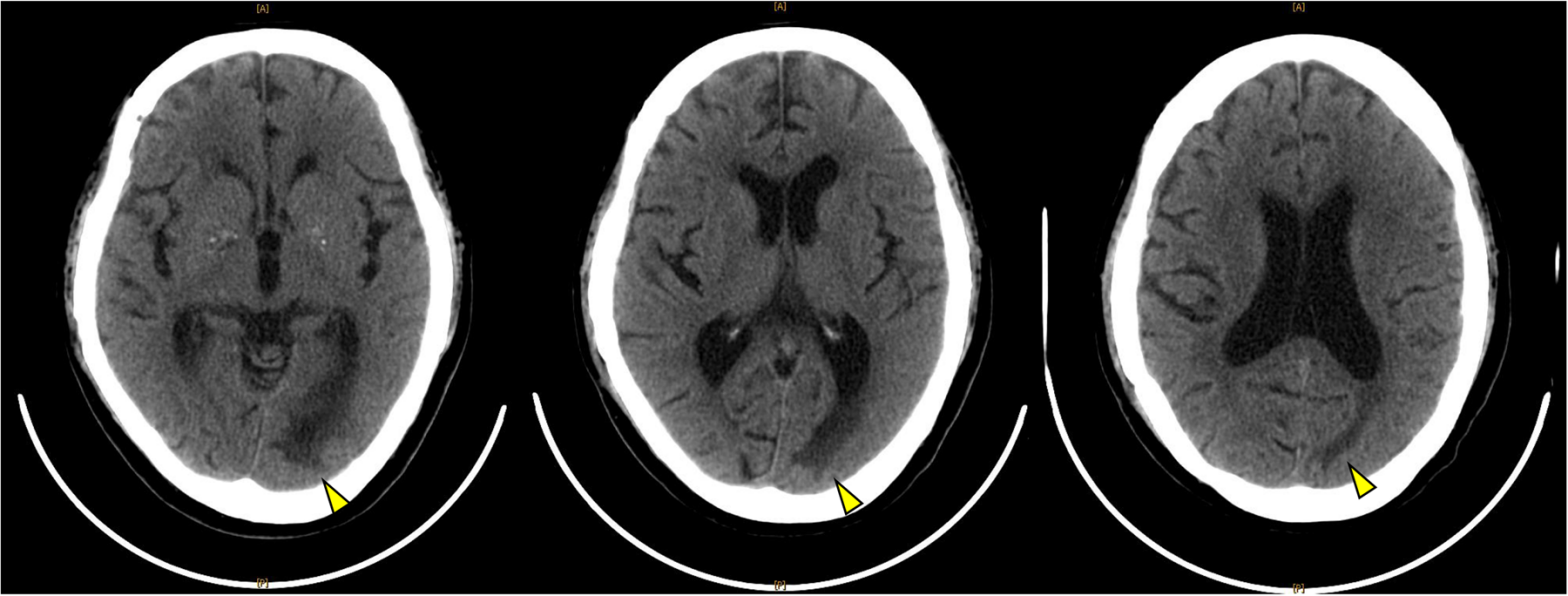
Head computed tomography performed after the onset of aphasic status epilepticus. Points indicate a low‐density lesion around the posterior horn of the lateral ventricle, which was considered a sequela of the traumatic cerebral contusion.

**Figure 2:**
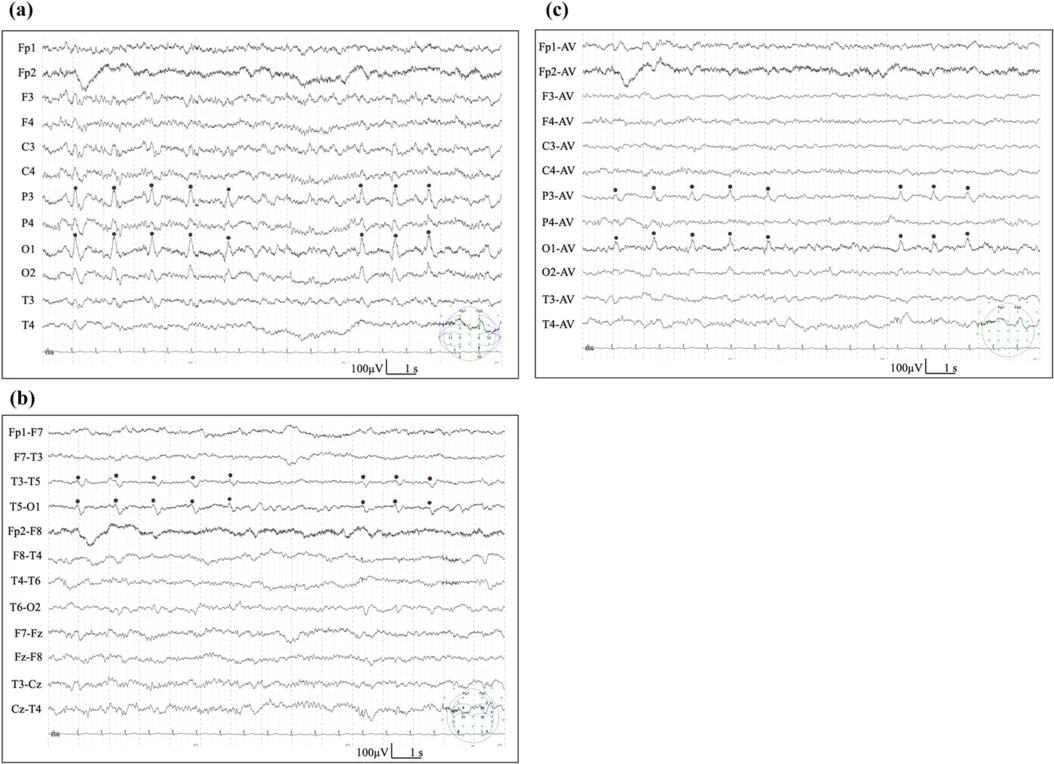
Initial electroencephalogram demonstrating left‐dominant periodic lateralized epileptiform discharges. (a) Unipolar montage. (b) Bipolar montage. (c) Average‐reference montage. Points indicate representative periodic lateralized epileptiform discharges.

**Figure 3:**
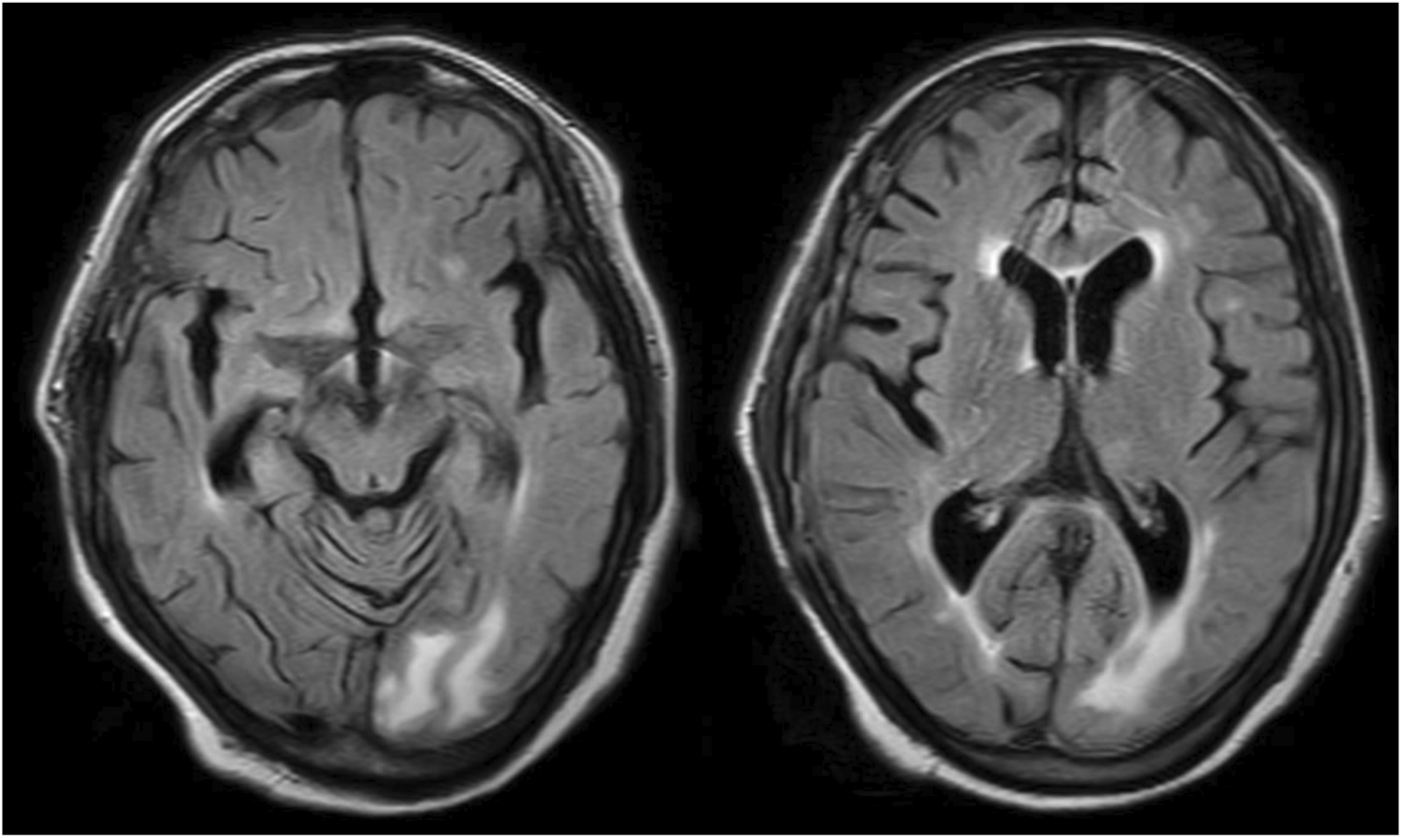
Brain magnetic resonance imaging performed after the onset of aphasic status epilepticus.

At readmission, she exhibited marked oversedation, which was considered to be an adverse effect of perampanel. Perampanel was therefore gradually tapered and discontinued by Day 135 after the initial seizure, and lacosamide was introduced and increased to 300 mg/day by Day 147 after the initial seizure. Thereafter, the oversedation resolved, and the patient's aphasia, agitation, and irritability disappeared, allowing normal communication. However, on Day 167 after the initial seizure, sensory aphasia, agitation, and irritability recurred abruptly. No qualitative difference in the symptoms from the initial ASE was observed. EEG performed at that time again demonstrated periodic lateralized epileptiform discharges (Figure 4). A recurrence of ASE was diagnosed, and intravenous diazepam (10 mg) was administered. Following treatment, the EEG abnormalities resolved, and the aphasia and irritability rapidly disappeared. Levetiracetam was subsequently initiated and gradually increased to 2000 mg/day. No further aphasic seizures or irritability occurred during hospitalization, allowing risperidone to be gradually discontinued. Mini‐Mental State Examination performed on Day 232 after the initial seizure scored 26/30 with 4 points lost during the assessment, and the Frontal Assessment Battery completed on the same day scored 15/18. After completing rehabilitation and discharge planning, the patient was discharged home on Day 242 after the initial seizure. Figure 5 shows a schematic timeline illustrating the inpatient clinical course.

**Figure 4:**
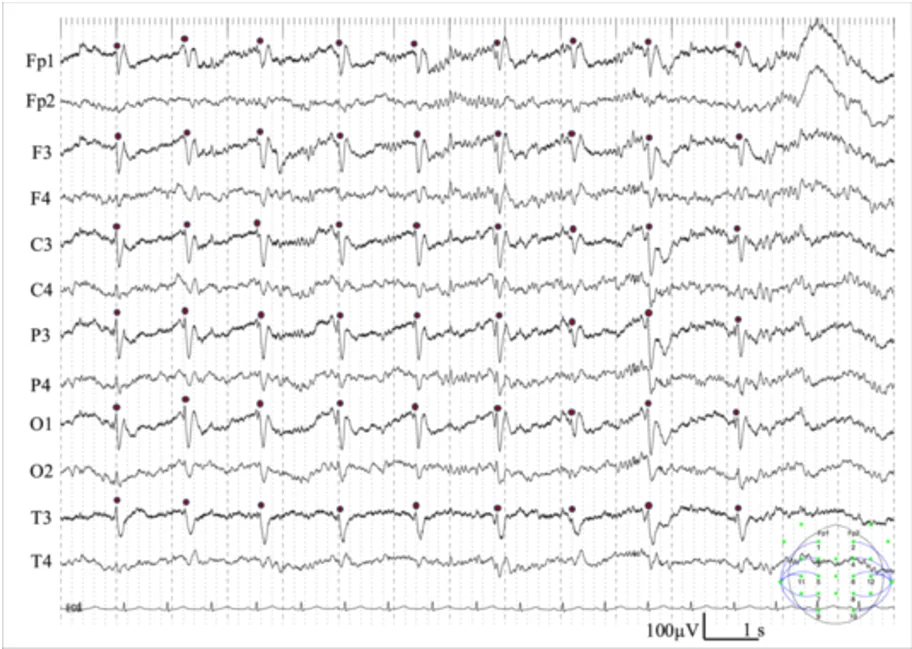
Electroencephalogram obtained on Day 167 demonstrating left‐dominant periodic lateralized epileptiform discharges. Points indicate left‐dominant periodic lateralized epileptiform discharges.

**Figure 5:**
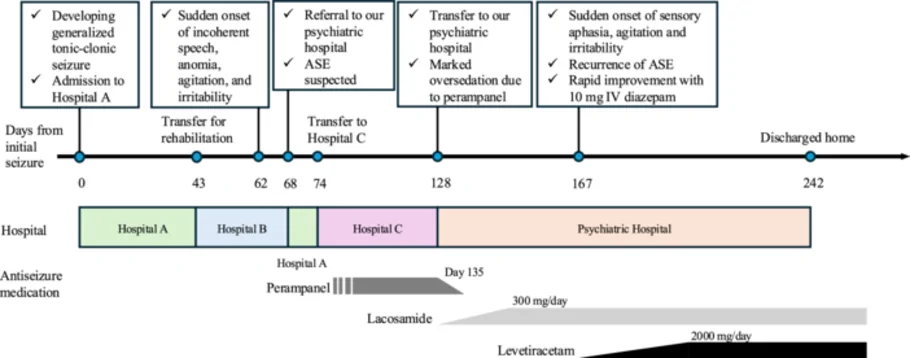
A schematic timeline illustrating the inpatient clinical course. Abbreviation: ASE, aphasic status epilepticus; IV, intravenous.

Following discharge, episodes of aphasia accompanied by agitation recurred approximately once every 2 months. Ultimately, combination therapy with levetiracetam (3000 mg/day), lacosamide (400 mg/day), and clonazepam (1 mg/day) achieved complete remission of the aphasic seizures, agitation, and irritability, with no recurrence during more than 2 years of follow‐up. Additionally, no apparent cognitive impairment or changes to the level of engagement in daily activities were clinically observed.

## DISCUSSION

We reported an atypical case of ASE presenting predominantly with agitation and irritability, which was rapidly diagnosed by EEG on the basis of the patient's abrupt symptom onset and neurological history. Because prominent psychiatric symptoms dominated the initial clinical presentation, an epileptic etiology was not initially suspected before referral to a psychiatric hospital. In patients with ASE in which psychiatric symptoms predominate, the underlying epileptic disorder may be overlooked. Careful assessment of the temporal profile of symptom onset and relevant neurological history is therefore essential for recognizing a possible epileptic etiology and prompting timely EEG evaluation.

ASE accompanied by psychiatric symptoms may be particularly prone to delayed recognition of the underlying epileptic disorder and referral to psychiatric services. Aphasia is generally regarded as the principal manifestation of ASE, and disturbances of consciousness, behavioral changes, and psychiatric symptoms have traditionally been considered uncommon.^2^ Nevertheless, several cases have been reported in which psychiatric symptoms were the initial or predominant manifestations of ASE.^4^, ^5^, ^6^, ^7^ In one such case, failure to recognize the underlying epilepsy resulted in an initial diagnosis of schizophrenia.^7^ Furthermore, patients with sensory aphasia and preserved speech fluency may be mistakenly suspected of having a primary psychotic disorder because of their incoherent speech.^7^ In the present case, the combination of prominent psychiatric symptoms and sensory aphasia may have delayed the consideration of an epileptic etiology before referral to our hospital.

In our patient, consideration of the abrupt symptom onset together with her history of traumatic brain injury and generalized tonic–clonic seizure prompted EEG evaluation, resulting in the early diagnosis of ASE at a psychiatric hospital. ASE is an uncommon condition and is frequently underrecognized or diagnosed late.^3^ The present case suggests that its diagnosis may be particularly challenging when psychiatric symptoms predominate, because the differentiation of ASE from primary psychiatric disorders or delirium becomes necessary. To differentiate from these disorders, acute‐onset psychiatric symptoms are an important clinical clue suggesting an epileptic etiology.^8^, ^9^ Therefore, careful attention to the temporal profile of psychiatric symptoms, together with an epileptogenic background, should prompt consideration of EEG and may facilitate the early diagnosis of the underlying neurological disorder.

The possible contribution of alcohol use should also be considered in this case. Given the history of alcohol use disorder, the initial generalized tonic–clonic seizure may have been related to alcohol withdrawal rather than solely due to head injury. However, the subsequent ASE developed after a prolonged period of hospitalization without alcohol consumption, making acute alcohol withdrawal an unlikely explanation for the aphasic seizures. In addition, there were no clinical or neuroimaging findings indicative of Wernicke encephalopathy. Although ASE has been reported in patients with alcohol use disorder,^2^, ^10^ alcohol‐related brain damage has not been established as a specific precipitating factor for ASE. Further evidence is needed to better understand the potential association between alcohol use disorder, alcohol‐related brain damage, and ASE.

## CONCLUSION

We reported a case of ASE presenting with agitation and irritability. In atypical presentations of ASE in which psychiatric symptoms predominate, the underlying epileptic disorder may be overlooked, leading to referral for psychiatric evaluation. Careful assessment of the temporal profile of symptom onset and relevant neurological history is essential for recognizing a possible epileptic etiology and prompting timely EEG evaluation.

## AUTHOR CONTRIBUTIONS

Yuto Yamada and Shigeru Takahashi contributed to the patient's treatment. Yuto Yamada and Masaki Fujiwara wrote the first draft. All authors contributed to the discussion of the patient's symptoms and approved the final version of the manuscript.

## CONFLICT OF INTEREST STATEMENT

Yuto Yamada has received honoraria from Otsuka, Daiichi Sankyo, Sumitomo Pharma, Shionogi, Takeda, and Lundbeck. Masaki Fujiwara has received honoraria from Eisai. Shigeru Takahashi has nothing to disclose.

## ETHICS APPROVAL STATEMENT

Not applicable.

## PATIENT CONSENT STATEMENT

Written informed consent was obtained from the patient to publish the case report.

## CLINICAL TRIAL REGISTRATION

Not applicable.

## Data Availability

Not applicable.
